# Costs associated with treatment of insomnia in Alzheimer’s disease caregivers: a comparison of mindfulness meditation and cognitive behavioral therapy for insomnia

**DOI:** 10.1186/s12913-022-07619-w

**Published:** 2022-02-19

**Authors:** Tanya G. K. Bentley, Daisy Castillo, Nina Sadeghi, Dominique Piber, Judith Carroll, Richard Olmstead, Michael R. Irwin

**Affiliations:** 1 Cousins Center for Psychoneuroimmunology, Jane and Terry Semel Institute for Neuroscience and Human Behavior, University of California, Los Angeles, USA; 2 Department of Psychiatry and Biobehavioral Sciences, David Geffen School of Medicine, University of California, Los Angeles, California USA; 3 Department of Psychiatry, Charité – Universitätsmedizin Berlin, Berlin, Germany

**Keywords:** Insomnia treatment, Alzheimer disease, Caregivers, Mindfulness, Cognitive behavioral therapy for insomnia, Cost analysis

## Abstract

**Background:**

Among the over 5 million informal caregivers for patients with Alzheimer’s disease (AD) in the United States (US), over 60% experience insomnia. Research on insomnia treatment efficacy in AD caregivers is limited. An ongoing randomized non-inferiority clinical trial, the Caregiver Sleep Research study, is evaluating whether mindfulness meditation is non-inferior to cognitive behavioral therapy for insomnia (CBT-I) in the treatment of insomnia in AD caregivers. The present report examines estimated intervention costs in this ongoing trial.

**Methods:**

Micro-costing was used to itemize and abstract costs of the two interventions: a mindfulness-based intervention known as mindful awareness practices for insomnia (MAP-I); and CBT-I. This approach involves collecting detailed data on resources utilized and the unit costs of those resources, thereby revealing actual resource use and economic costs for each treatment arm. Personnel time, patient time, and supplies were inventoried, and unit costs were applied. Caregiver time costs, including travel, were based on US Labor Bureau home-health aide national mean hourly wages; instructor/staff costs were based on hourly wages. Per-participant and program costs were calculated assuming individual- and group-delivery to reflect real-world implementation. Sensitivity analyses evaluated robustness of estimates.

**Results:**

From the societal perspective, per-participant MAP-I costs were $1884 for individual and $1377 for group delivery; for CBT-I, these costs were $3978 and $1981, respectively. Compared with CBT-I, MAP-I provided cost savings of $2094 (53%) and $604 (30%) per treated caregiver for individual and group delivery, respectively. From the US healthcare system perspective, MAP-I vs. CBT-I participant savings were $1872 (65%) for individual and $382 (44%) for group interventions, respectively. For MAP-I and CBT-I, instructor in-class time was the highest cost component. Results were most sensitive to combined instructor time costs.

**Conclusions:**

Treatment of insomnia with MAP-I, compared to CBT-I, yields substantial cost savings for society and the healthcare system. With this potential for cost savings, results of the ongoing non-inferiority trial have critical implications for insomnia treatment dissemination and its benefits to AD caregivers and other community populations with insomnia.

**Supplementary Information:**

The online version contains supplementary material available at 10.1186/s12913-022-07619-w.

## Background

Over 5 million Americans provide informal caregiving to individuals with Alzheimer’s disease (AD) [1]. This number is predicted to increase 3-fold over the next decade given expected increases in AD prevalence [2]. Informal caregiving negatively impacts caregivers’ mental and physical health, increasing mortality risk by 63% compared with non-caregiving controls [1, 2]. Caregivers are more likely to suffer from insomnia, mood and anxiety disorders, substance abuse, and chronic medical disorders, and have 14% higher average annual healthcare costs than matched controls [3]. The value of unpaid, informal caregiving for individuals with AD and other dementias in 2019 was estimated at $244 billion [4, 5].

Insomnia occurs in over 60% of AD caregivers and has independent negative influences on caregiver health and mechanisms of disease risk [6–10]. Insomnia is characterized by difficulty falling or staying asleep, waking repeatedly throughout the night, and experiencing non-restorative sleep, resulting in daytime impairments such as fatigue and depressed mood [11]. The increased prevalence of insomnia in AD caregivers is thought to be related to psychological distress of caregiving, and possibly to circadian rhythm disturbance in the AD patient [10, 12–14]. In older adults including AD caregivers, insomnia is associated with increased risks of chronic disease and death [5, 15–17].

The economic burden of insomnia is significant. Direct costs of insomnia include costs of added healthcare utilization, such as office visits, medication costs, and testing; indirect costs include the costs of insomnia-induced lost resources such as absenteeism, presenteeism, or accidents. Insomnia-related comorbidities may increase healthcare costs by up to 80% [18], with total costs of untreated insomnia as high as $100 billion per year in the US [18].

Over 120 intervention trials have been conducted in AD caregivers, yet no prior study has targeted insomnia disorder in this population [2, 5, 19], even though insomnia can be effectively treated. While sedative medications, such as benzodiazepines and non-benzodiazepine-hypnotics, are often used to treat insomnia, they hold risks for dependence and have adverse daytime cognitive effects especially in older adults [20]. Additionally, such medications would not be recommended for AD caregivers who might need to respond to the AD patient during the night. Cognitive behavioral therapy for insomnia (CBT-I) is currently considered the treatment of choice for insomnia by the American Academy of Sleep Medicine and American College of Physicians [21, 22]. However, CBT-I is not widely disseminated, because it is intensive, requires administration by highly trained therapists, and is often not available in primary- or collaborative-care settings [23]. Hence, limitations of current treatment for insomnia highlight the need for community-accessible treatments that can improve insomnia.

Less is known about the efficacy of alternative behavioral treatments, although mindfulness-based interventions (MBIs) hold the potential to meet the needs of community-dwelling older adults such as AD caregivers who have insomnia. Indeed we have found that a mindfulness-based intervention, mindful awareness practices for insomnia (MAP-I) can improve sleep quality in older adults with an effect size comparable to CBT-I [24]. MAP-I, an insomnia-tailored version of MAP, is a validated and curriculum-based MBI that trains one in the systematic practice of attending to moment-by-moment experiences, thoughts, and emotions from a nonjudgmental perspective [5, 25, 26], similar to mindfulness-based stress reduction. However, in contrast to mindfulness-based stress reduction, MAP and MAP-I are more accessible by not requiring a day-long retreat or the learning and practice of hatha yoga. Moreover, MAP-I is designed to target insomnia by incorporating practice prior to bed, use of practice in the bed during night-time awakenings, and daily body scans, which together have been found to hasten sleep onset and improve sleep efficiency, sleep quality and daytime function [24]. MAP-I holds promise in addressing the specific needs of AD caregivers, as this “outside of the clinic” treatment may also reduce stress perceptions that precipitate insomnia and perpetuate its persistence in caregivers [27].

Although there has been a recent upsurge in research on the effectiveness of mindfulness-based interventions [27], very few studies have evaluated the costs of these interventions. Economic analyses are critical for guiding resource allocation decisions within the constraints of limited healthcare resources. To the best of our knowledge, no prior costing nor cost-effectiveness analyses have been conducted on any mindfulness-based intervention for insomnia, either in general or specifically in the AD caregiver population. As implementation of either the MAP-I or CBT-I interventions in real-world settings will be highly dependent on implementation costs, there is an important need to evaluate their comparative costs.

An ongoing non-inferiority trial, Caregiver Sleep Research (CARES), is evaluating whether MAP-I is non-inferior to CBT-I for the treatment of insomnia in AD caregivers (NCT03538574). Here, we use micro-costing to measure and estimate the costs of implementing the MAP-I and CBT-I interventions in AD caregivers enrolled in CARES. Specifically, this study aims to measure the resource utilization associated with implementing the MAP-I and CBT-I interventions in the context of the CARES clinical trial; we estimated the costs associated with such utilization and compared total and per-participant costs of MAP-I and CBT-I for AD caregiver insomnia treatment when considering either individually- or group-delivered interventions. Micro-costing is a cost estimation method that involves “direct enumeration and costing-out of every input consumed in treatment of each particular patient,” and has been recommended by the US Panel on Cost Effectiveness in Health and Medicine as the preferred approach to cost estimation [28]. By collecting detailed data on resources utilized and the unit costs of those resources, the micro-costing process reveals actual resource use and economic costs. It is particularly useful for estimating costs associated with new interventions and when compared with status-quo or standard care approaches. Micro-costing is most accurate when resource consumption is tracked as it occurs, allowing the extraction of exact data on the types and quantities of resources used.

Given the more intensive trainings and practice requirements of CBT-I compared with MAP-I, it was hypothesized that MAP-I would use fewer resources and have lower total and per-patient costs than CBT-I. If the results of the CARES non-inferiority trial show that MAP-I has equivalent or greater effect on insomnia outcomes compared with CBT-I, and MAP-I is also found to be of equivalent or lower cost, this research could have broad implications for changing the standard of care treatment for insomnia and reducing the staggering costs of AD caregiver burden for the individual, family, healthcare, and society. Evaluating the comparative costs of these 2 interventions will, for the AD caregiver population, inform insomnia treatment decision-making and implementation in real-world settings. Additionally, cost-benefit analyses based on these findings and those from the CARES trial will be useful in guiding methodology for estimating costs alongside other mindfulness-based interventional trials.

## Methods

### Recruitment, sample and interventions of randomized controlled trial

The ongoing CARES trial is a partially single-blind, parallel non-inferiority trial evaluating the non-inferiority of MAP-I compared with CBT-I for treating insomnia in AD caregivers with insomnia over a one-year follow-up. Protocol information can be found in the registration document (ClinicalTrials.gov: NCT03538574). Briefly, the sample eligible criteria for CARES include AD spousal and family member caregivers (40–85 years) who are self-identified as the principal person taking care of the AD patient with a physician-based diagnosis of AD, and who are devoting at least 1 hour daily to the care of the AD patient. Diagnostic interview data were obtained by the Structured Clinical Interview for Diagnosis and by Duke Structured Interview for Sleep Disorders to ascertain the presence of insomnia using Diagnostic and Statistical Manual-5 (DSM-5) criteria; all participants had DSM-5 insomnia disorder.

The primary outcome is change in insomnia severity as measured by the Insomnia Severity Index (ISI), [29] with assessment of secondary sleep outcomes including clinical response, remission of insomnia disorder, and change in sleep-related daytime dysfunction such as depressive symptoms and fatigue. Morin et al. [29] have evaluated what decrease in insomnia severity as measured by the ISI is clinically meaningful. An ISI score change of 8 or more indicates a moderate clinical improvement, and a change of 8 on the ISI is a minimally important difference in insomnia treatment among cancer survivors [30]. An ISI change of 9 or more optimally identifies participants with marked improvements relative to a rating of clinical global improvement as independently assessed. Marked insomnia improvement is anchored by complete or nearly complete symptom remission.

The caregiver population of interest is AD spousal and family member caregivers. Whereas emphasis on a homogeneous diagnostic group such as AD caregivers may limit generalizability, there are advantages for inclusion of only AD caregivers. Caregivers of individuals with different diseases may result in differences in caregiver burden, as well as differences in the precipitating and perpetuating factors related to insomnia, which together might complicate treatment effects.

Targeted sampling methods are used to identify AD caregivers residing within 15 miles of the University of California Los Angeles (UCLA) Westwood campus. Specifically, AD or other dementia patients who are enrolled in the UCLA Health System are identified by the Clinical and Translational Science Institute Informatics Program with extraction of patient demographics, contact information, diagnoses, problem list, and social history. An introductory letter and brochure describing the study are mailed to the AD patient household, with a follow-up eligibility interview conducted by phone.

### Trial procedures

The randomization sequence was generated via computerized random number generator in blocks of 7–10 participants in MAP-I and CBT-I (1:1) by a statistician who did not view participant data prior to allocation. Allocation concealment was maintained by the fact that no research staff had access to the allocation sequence. This sequence was recorded on sequentially numbered, opaque, and sealed envelopes. Assessors were blind to allocation*.*

### Randomized controlled trial interventions

Both MAP-I and CBT-I interventions are implemented in weekly, 120-min group sessions led by either MAP-I or CBT-I trained instructors over a 6-week treatment period. Both MAP-I and CBT-I sessions are conducted in small groups of approximately 7 caregivers. Following the 6-week intervention period, booster sessions, each lasting 120-min, are delivered at 2, 4, 6, 8, and 10 months follow-up. Booster sessions are implemented to address strategies to maintain adherence given the changes in the clinical status of the AD patient and the impact of changes in the AD patient’s sleep-wake activity on the AD caregivers and their sleep.

### Mindful awareness practices-insomnia (MAP-I)

MAPs focuses on the practice of mindfulness and its application in everyday life [25]. MAPs is a validated and curriculum-based mindfulness based intervention similar to mindfulness based stress reduction, with the exception that MAPs does not include a day-long retreat or hatha yoga and hence takes a more practical and accessible approach that focuses specifically on the practice of mindfulness and its application in everyday life. MAP-I is a modified version of MAPs that incorporates practice prior to bed, use of practice in the bed during night-time awakenings, and daily body scans. MAP-I may be especially valuable for AD caregivers who are awakened by the irregular sleep/wake routines of the AD patient, [13, 31] especially given that it can be practiced in the bedroom or bed prior to sleep. In our prior study, [24] MAP-I was hypothesized to reduce stress perceptions that precipitate insomnia and perpetuate its persistence in caregivers [27]. Indeed, by incorporating practice prior to bed, use of practice in the bed during night-time awakenings, and the daily body scan, MAP-I has been found to hasten sleep onset and improve sleep efficiency, sleep quality and daytime function [24].

Hence, MAP-I focuses on development of bodily awareness, formal meditation practices, and strategies for the daily informal use of mindfulness. Participants are instructed to practice mindfulness techniques at home on a daily basis, beginning with 5 minutes per day and increasing to 20 minutes per day over the 6-week period, including practice prior to bedtime. Moreover, participants are asked to record times of meditation practice in diaries each day. In addition to teaching mindfulness, the instructor works with participants to assess understanding of mindfulness practice and resolve issues that could interfere with home practice adherence, especially in relation to the evolving needs of the AD patients. Participants are also provided with a book on mindfulness and homework materials to guide daily adherence to mindfulness practice. MAP-I sessions are weekly for 120 minutes per session over a 6-week treatment period, for 6 total sessions.

### Cognitive behavior therapy-insomnia (CBT-I)

CBT-I is a multicomponent (i.e., behavioral, cognitive, educational) intervention as previously described by Morin et al. [32]. CBT-I contains five validated components: cognitive therapy, stimulus control, sleep restriction, sleep hygiene, and relaxation, which together target sleep-related physiologic and cognitive arousal to reestablish restorative sleep function [32]. The content of the intervention is organized around a series of modules that is presented to patients in manualized form. CBT-I includes five treatment modules: (1) *Cognitive Therapy* uses cognitive restructuring principles to help patients identify maladaptive sleep cognitions, neutralize their effect, and facilitate more adaptive thinking about sleep and its importance, including training in other cognitive coping strategies such as relaxing self-talk, imagery, and distraction methods (e.g., repetition of a calming phrase, thought). (2) *Stimulus Control* targets sleep behavior directly by instructing patients to go to bed only when sleepy; use the bed only for sleep and sexual activity and not for other behaviors that compete with sleep; leave the bedroom after being unable to fall asleep within 20 minutes; repeat this process as often as necessary either before falling asleep or after awakening from sleep; and establish and adhere to a fixed time of arising each morning. (3) *Sleep restriction* limits the amount of time spent in bed to the amount of time spent sleeping. This method creates a mild state of sleep deprivation and is thought to facilitate a faster sleep onset and greater sleep continuity and quality. Specifically, the window of time available to sleep is altered weekly to approximate a sleep efficiency goal of 85%. Sleep efficiency is monitored weekly by the sleep diary, and when sleep efficiency is less than 80%, the sleep window is decreased by 15 to 20 minutes per night. The sleep window was not decreased to less than 5 hours per night because this level of restriction can lead to daytime drowsiness and related adverse events, which were monitored in the clinical trial during treatment as described below. (4) *Sleep hygiene* provides sleep education and discussion of the role of biological, psychological, social, and behavioral factors that affect sleep, such as stress, cognitive arousal, poor sleep hygiene, and mood disturbance. (5) *Relaxation* assists patients in developing behavioral goals in areas where sleep has disrupted their functioning and mood (e.g., work, social, physical activity), with the use of self-rewards (e.g., leisure, resting, relaxation), scheduling of pleasant events and mental exercises to increase relaxation, and awareness of positive emotional states. Addressing relaxation and mood throughout the protocol in an integrated manner is believed to augment the efficacy of the intervention in those who have high rates of stress such as caregivers with sleep disturbance, and also contribute to the maintenance and generalization of the intervention during follow-up. CBT-I sessions are weekly for 120 minutes per session over a 6-week treatment period, for a total of 6 sessions.

### Micro-costing methodology

Micro-costing was used to itemize and abstract costs of all components included in each of the two interventions, and thereby to estimate total and per-participant costs per intervention. These micro-costing methods are grounded in economic theory and based on the Panel’s recommendations, adapted for use with this specific intervention [28]. Specifically, we followed the 3-step approach outlined by the Panel: identification; measurement; and valuation of resources used. For each step in each intervention arm, all inputs – including personnel time, patient time, supplies and equipment – were inventoried and measured. Unit costs were applied to the quantities of each such resource consumed, and results were summed to obtain total costs per component and overall for MAP-I and CBT-I interventions. Per-participant costs were estimated by dividing total costs by number of participants. Sensitivity analyses were conducted to evaluate the robustness of estimates to variations in key cost components.

The Panel recommends that any kind of economic analysis be conducted from the societal as well as other relevant perspectives. A societal perspective incorporates costs to patients as well as providers and other members of the healthcare system. In this study, our base case analysis was conducted from the societal perspective, including both indirect costs faced by caregivers, such as their time and travel for doing the intervention, and direct costs faced by the healthcare system, such as costs of provider and staff time and materials needed for implementing the interventions. The Panel also recommends that analyses be presented from other perspectives as appropriate. To provide cost estimates that are informative to providers, policymakers, and patients alike, we present in sensitivity analyses results when considering the healthcare system’s perspective.

The analysis considered only resources necessary for reproducing the specific interventions and not the research-specific costs of reproducing the clinical trial itself, which would not be applicable for real-world MAP-I or CBT-I implementation. For example, we excluded costs involved in communicating with funding agencies, collecting clinical trial specific data, conducting analyses, and disseminating study findings.

### Step 1: identifying intervention components, enumerating resources

The first phase of micro-costing analysis involves identifying and thoroughly delineating all resources needed for developing and implementing the interventions. Resources, and subsequently their associated costs, are divided into three categories: intervention set-up resources; time-dependent intervention resources; and variable intervention resources. Categorizing costs in this way allows us to extrapolate findings to interventions or programs of different sizes. Intervention set-up resources include those required for preparing the intervention prior to implementation, comprising for this analysis: staff and instructor training; staff time in session planning, preparing materials, participant scheduling, and room set-up and clean-up; and fixed equipment. Materials prepared by staff comprised handouts, practice diaries (MAP-I), worry logs and sleep diaries (CBT-I), adherence questionnaires, and slides (CBT-I).

Time-dependent resources are those required for conducting the intervention, are incurred for as long as the intervention is running, and are independent of the number of participants in the program. This analysis included the following time-dependent resources: staff time for materials distribution and participant attendance tracking; for MAP-I, staff time tracking meditation diaries and practice; for CBT-I, staff time recording data from sleep diaries and calculating sleep efficiency; and instructor time in class, planning CBT-I treatments based on sleep efficiency calculations, and with participants outside of class time reviewing treatment plans and/or answering questions. Because these staff and instructor time resources were estimated as overall program-specific resources that were ongoing throughout the course of the CARES study, they were included as time-dependent resources rather than variable. However, since cost results are presented per-participant, costs associated with instructor and staff time can be estimated as a variable intervention component as needed by users of this analysis. Overhead and facilities were estimated to be minimal and similar between the two interventions and thus their associated costs were excluded from the analysis.

Variable program resources are those that vary with the number of participants. Variable resources comprise participant time, materials for individual participants, and staff or instructor time with individual participants. The following variable resources were included in this analysis: time for a caregiver separate from the study participant to care for the AD patient while the study participant travels to/from and participates in the intervention; variable equipment costs of homework materials for each participant in both interventions; and participant time in sessions, traveling to/from sessions, and completing home components such as sleep diaries, worry logs, or home meditation practice.

We specified these cost components for each of the MAP-I and CBT-I interventions based on a detailed review of all work steps involved in program set-up and implementation. Tracking forms, staff interviews, and data from the interventions were used to determine the components in each category. For each component, we determined the measurement unit to be used (e.g., hours + fraction of hours for staff/instructor/participant time; average hours for transportation costs) and the method by which each resource would be valued (e.g., hourly wage rates or salary + benefits for time costs; purchase costs for materials; etc.). All included components by category, their measurement units and valuations are shown in Table 1.

**Table 1:** Resource utilization: measured units and cost valuation

RESOURCE	MEASURED UNITS	COST VALUATION
**INTERVENTION SET-UP RESOURCES**		
Instructor Training		
Time	Hours	Salary + benefits
Courses, Exams	Items	Purchased cost
Staff Training	Hours	Salary + benefits
Session Planning	Hours	Salary + benefits
Materials Preparation	Hours	Salary + benefits
Participant Scheduling	Hours	Salary + benefits
Room Set-Up, Clean-Up	Hours	Salary + benefits
Fixed Equipment (meditation bell, computer)	Items	Purchased cost
**TIME-DEPENDENT RESOURCES**		
Staff Time		
Materials Distribution	Hours	Salary + benefits
Session Tracking	Hours	Salary + benefits
Calculating Sleep Efficiency	Hours	Salary + benefits
Instructor Time		
In-Class Time	Hours	Salary + benefits
Treatment Planning	Hours	Salary + benefits
Non-Structured Time with Participants	Hours	Salary + benefits
Overhead and Facilities	N/A^a^	N/A^a^
**VARIABLE RESOURCES**		
Other AD Caregiver Time while Participant in Intervention	Hours	Hourly wage rate
Homework Materials	Item	Purchased cost
Participant Time		
In Sessions	Hours	Hourly wage rate
Travel To/From Sessions	Average hours	Hourly wage rate
Completing Home Components (E.g., sleep diaries, worry logs, home meditation practice)	Hours	Hourly wage rate

*N/A* Not applicable, *CBT-I* Cognitive behavioral therapy for insomnia, *MAP-I* Mindful awareness practices for insomnia

^a^Overhead and facilities costs were estimated to be minimal and similar between the two interventions, thus were excluded from the analysis

### Step 2: measuring resource use

The next phase of this analysis involved estimating resource utilization associated with each of the above intervention components. We measured inputs real-time while the CARES trial was underway to increase the reliability and validity of the cost data. Detailed record analysis was conducted to determine quantity of materials and time used during the interventions. Supplemental interviews were performed as needed with intervention staff regarding any additional resources and personnel time used for delivering the interventions.

We recorded all utilization for each of the resource components outlined above for the MAP-I and CBT-I interventions. Instructor and staff training resources included program-specific training prior to program initiation considered necessary for successfully intervention implementation. MAP-I and CBT-I instructor training requires specific curricula, each with different time, course, and/or exam requirements; component and cost details for each are included in Supplemental Table 1a-b (see Additional File 1). CBT-I assumed the use of CBT-I-trained psychologists, whose training comprises some components that are optional for certification. As such, costs for this training were estimated considering a range of training approaches and, assuming that these approaches would be implemented with equal likelihood across instructors, the average costs of all approaches was used for this analysis. For intervention staff and coordinators, intervention-specific training did not differ between interventions, was minimal or nonexistent, and was assumed to be zero for both interventions. Participant travel to/from the intervention location were estimated based on participant time and not based on mileage; average travel time included round-trip driving to the study’s institution assuming that participants were traveling from an average 25-mile radius around the institution. Details of all human resource time utilization are included in Supplemental Table 2a-c (see Additional File 2).

### Step 3: valuing resources

The final phase of the micro-costing analysis involved assigning monetary values to each of the inputs determined in Step 2 and multiplying those values by the units of resource utilization for each component in the two interventions. This produced cost estimates overall and costs per component. All resource unit costs used as inputs in this analysis are shown in Table 2.

**Table 2:** Resource cost inputs, 2020 US dollars^a^

Resource	MAP-I	CBT-I
FTE Hourly Rates^b^		
Instructors	$27	$82
Research assistant	$34	
Project manager	$46	
Participants	$12	
Other AD caregivers^c^	$12	
Other Intervention Set-up Costs		
Instructor Training^d^		
Courses	$7200	$800
Manuals, materials	–	$200
Supervisor time^e^	–	$700
Fellowships		$575
Retreats	$4000	–
Exams	–	$250
Fixed Equipment		
Meditation bell	$23	–
Laptop computer	–	$313
Other Variable Resource Costs		
Compact disc (each)	$1	

*CBT-I* Cognitive behavioral therapy for insomnia, *FTE* Full-time equivalent, *MAP-I* Mindful awareness practices for insomnia

^a^Hourly FTE rates rounded to nearest $1, all other values rounded to nearest $10. Time-dependent resources not listed in this table because only human resource time (estimated using FTE hourly rates) is included in this category

^b^Based on annual salary + fringe benefits, assuming a 2088-h work year

^c^AD caregivers separate from study participants to care for the AD patients while study participants travel to/from and participate in the intervention

^d^Not including instructor training time

^e^To sit for the exam, the Society of Behavioral Sleep Medicine recommends 250 h of behavioral sleep medicine work under consultation with an expert, with at least 1 h/week of supervisor consultation; assuming 40-h weeks equates with a minimum of 6.25 direct supervision cases which are rounded up to 7 to avoid person-divisibility and assuming supervisor hourly FTE rate of $100

Unit costs were estimated based on several approaches. Instructor and staff costs for time spent on the interventions were valued as per-hour salary costs from annual salary and benefits, assuming a 2088-hour work year and rounding study tasks up to the nearest 5 minutes. Two different CBT-I therapists who were equally trained and monitored to treatment fidelity performed CBT-I over the duration of the clinical trial, with each taking on different treatment groups. Because these two therapists had different salaries, their average salary was used for the final CBT-I therapist cost. Costs of materials and supplies used in the interventions were obtained from research administrators. Fixed equipment costs were estimated based on “Amazon’s choice” prices for each item as of February 13, 2020.

Following a commonly-used approach in caregiver cost analyses, costs of participant caregivers’ time was estimated at $12.18 per hour based on the US Department of Labor’s national mean hourly wage rate for home health aides during the study period [33–35]. This assumes that their caregiving time costs are valued at the cost of lost wages for this type of job only, even though many if not all informal caregivers’ alternate jobs are likely not home health aide jobs and may provide salaries that are either higher or lower than the $12.18 estimate. It also assumes that the opportunity cost of caregivers’ leisure time lost is valued at the same rate as lost work time (i.e., productivity losses), even though some caregivers may value lost leisure time at a higher or lower rate than lost work time. The time spent by other caregivers paid to care for the participants’ AD patients while the participant traveled to/from and participated in the intervention was valued at the same rate.

Instructor training costs were estimated based on interviews with experts and the best available evidence regarding current costs of MAP-I and CBT-I certifications.

### Base case analyses

In the base case, MAP-I and CBT-I costs were calculated per-participant and per-program, including costs associated with both the 6 regular treatment sessions and the 5 followup booster sessions. Since both interventions are designed to be delivered in real-world settings either individually or in small groups, costs were estimated assuming both such approaches by excluding group-specific costs (e.g., room setup/cleanup) for individually-delivered programs. Data collection and analysis were conducted using Microsoft Excel software.

### Sensitivity analyses

To evaluate the impact on total costs of uncertainties in key cost categories, sensitivity analyses were conducted by excluding various resource components in one-way and multiple-way sensitivity analyses. Per-participant costs for each intervention and savings associated with MAP-I compared with CBT-I were estimated for each sensitivity analysis, considering both individually- and group-delivered programs. The following one-way sensitivity analyses were conducted: considering a US healthcare system perspective instead of a societal perspective by excluding indirect costs associated with participant time and other AD caregiver time; excluding instructor training costs, salary differences, and non-structured time (i.e., answering questions outside of session time); excluding participant travel time; and excluding booster sessions, fixed equipment, and other AD caregiver time. Two- and three-way sensitivity analyses were run excluding: all structured instructor time, both in sessions and in structured treatment plan development; all structured and non-structured instructor time; and all participant time, both in sessions and for travel to/from sessions.

## Results

### Total per-participant and program costs

Table 3 shows MAP-I and CBT-I per-participant and program costs for individually- and group-delivered interventions, presented from the societal perspective and by individual component, by component categories, and in total. All cost estimates are based on 2020 US dollars. From the societal perspective, MAP-I and CBT-I participant costs for individually-delivered interventions were $1884 and $3978, respectively, and were $1377 and $1981, respectively, for group-delivered interventions. These results translate into per-participant savings associated with using MAP-I of $2094 (53%) and $604 (30%) for individually- and group-delivered interventions, respectively. Assuming a 50-participant program, individually-delivered MAP-I and CBT-I programs cost $94,173 and $198,865, respectively, and $68,859 and $99,036 for group-delivered programs. Since program costs were based on summing individual component costs, rounding may cause slightly different cost estimates from those attained when multiplying per-participant total costs by 50 (participants).

**Table 3:** MAP-I and CBT-I societal costs, per-participant and per-program for individually- and group-delivered programs^a^

RESOURCE	INDIVIDUALLY-DELIVERED				GROUP-DELIVERED			
	MAP-I		CBT-I		MAP-I		CBT-I	
	Participant	Program	Participant	Program	Participant	Program	Participant	Program
**INTERVENTION SET-UP**								
Instructor Training								
Time	$66	$3300	$320	$15,975	$66	$3300	$320	$15,975
Courses, Exams	$224	$11,200	$51	$2527	$224	$11,200	$51	$2527
Staff Training	$0	$0	$0	$0	$0	$0	$0	$0
Session Planning	$1	$50	$6	$300	$18	$900	$23	$1150
Materials Preparation	$26	$1300	$26	$1300	$26	$1300	$26	$1300
Participant Scheduling	$11	$550	$16	$800	$11	$550	$16	$800
Room Set-Up, Clean-Up	$0	$0	$0	$0	$21	$1050	$23	$1150
Fixed Equipment	$1	$23	$6	$313	$1	$23	$6	$313
**Set-up Totals**	**$329**	**$16,423**	**$425**	**$21,215**	**$367**	**$18,323**	**$465**	**$23,215**
**TIME-DEPENDENT**								
Staff Time								
Materials Distribution	$17	$850	$30	$1500	$17	$850	$30	$1500
Session Tracking	$13	$650	$15	$750	$13	$650	$15	$750
Calculating Sleep Efficiency	$0	$0	$20	$1000	$0	$0	$20	$1000
Instructor Time								
In-Class Time	$594	$29,700	$1802	$90,100	$85	$4243	$257	$12,871
Treatment Planning	$0	$0	$328	$16,400	$0	$0	$47	$2343
Non-Structured Time with Participants	$41	$2050	$246	$12,300	$6	$293	$35	$1757
Overhead and Facilities^b^	$0	$0	$0	$0	$0	$0	$0	$0
**Time-dependent Totals**	**$665**	**$33,250**	**$2441**	**$122,050**	**$121**	**$6036**	**$404**	**$20,221**
**VARIABLE**								
Other AD Caregiving Time while Participant in Intervention	$402	$20,100	$426	$21,300	$402	$20,100	$426	$21,300
Homework Materials for Each Participant	$1	$50	$1	$50	$1	$50	$1	$50
Participant Time								
In Sessions	$268	$13,400	$293	$14,650	$268	$13,400	$293	$14,650
Travel To/From Sessions	$134	$6700	$134	$6700	$134	$6700	$134	$6700
Completing Outside-of-Session Components^c^	$85	$4250	$258	$12,900	$85	$4250	$258	$12,900
**Variable Totals**	**$890**	**$44,500**	**$1112**	**$55,600**	**$890**	**$44,500**	**$1112**	**$55,600**
**TOTALS**	**$1884**	**$94,173**	**$3978**	**$198,865**	**$1377**	**$68,859**	**$1981**	**$99,036**
**SAVINGS WITH MAP-I**	**$2094**	**$104,692**			**$604**	**$30,178**		

*N/A* Not applicable, *CBT-I* Cognitive behavioral therapy for insomnia, *MAP-I* Mindful awareness practices for insomnia

^a^Costs are presented per participant (“Participant”) and per program (“Program”). Individually-delivered programs assume that interventions are delivered to individuals in a 1:1 instructor-to-participant ratio; group-delivered assume group-based delivery in a 1:7 instructor-to-participant ratio

^b^Overhead and facilities costs were estimated to be minimal and similar between the two interventions, thus were excluded from the analysis

^c^E.g., sleep diaries, worry logs, home meditation practice

**Fig. 1:**
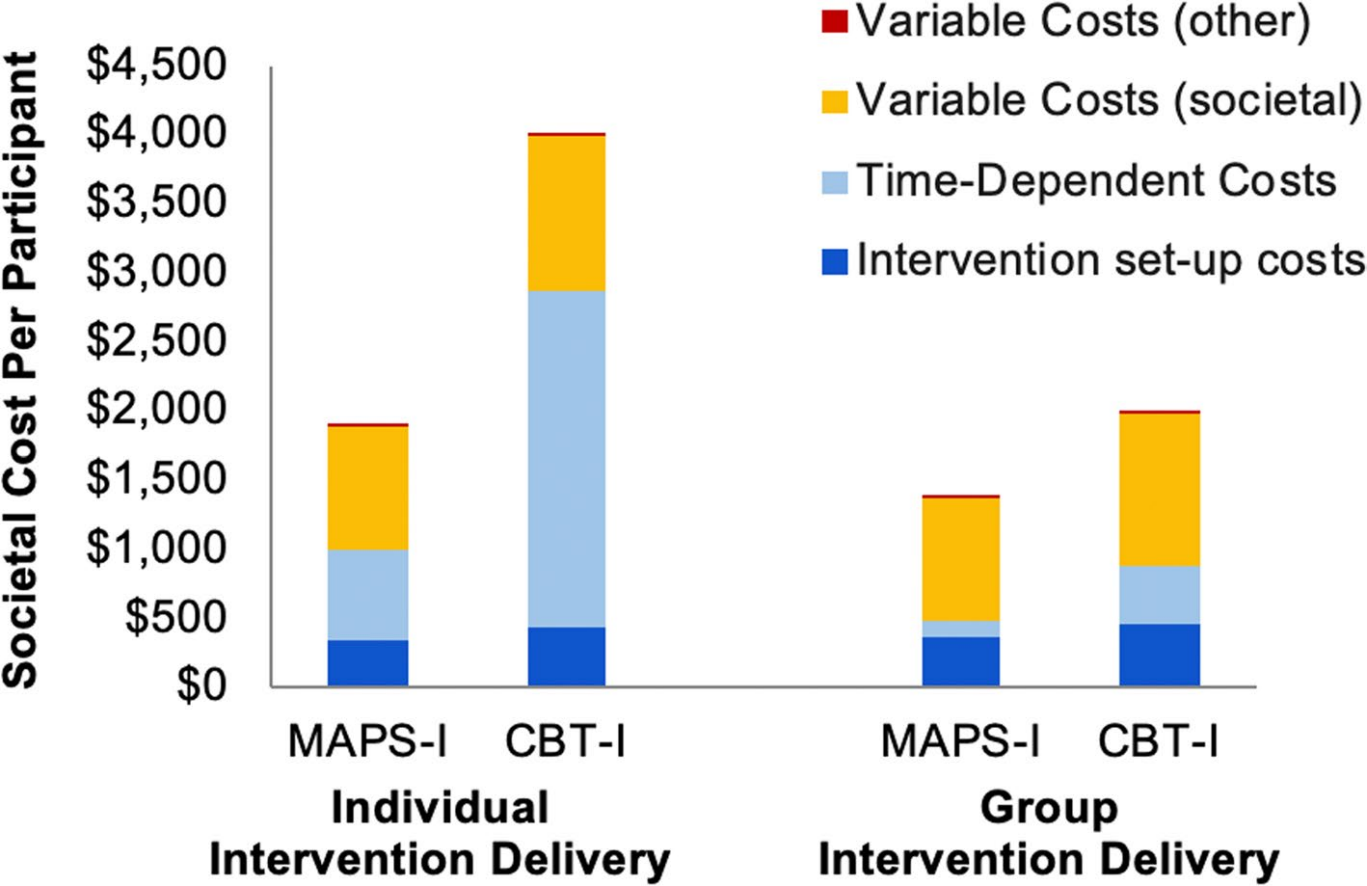
MAP-I and CBT-I participant costs by category, for individual and group delivery. Individual delivery assumes interventions delivered with a 1:1 instructor-to-participant ratio; group delivery assumes a 1:7 instructor-to-participant ratio; other variable costs were < $1. CBT-I, cognitive behavioral therapy for insomnia; MAP-I, mindful awareness practices for insomnia

CBT-I costs were higher than those for MAP-I under all scenarios and for both individually- and group-delivered interventions; this was the case in total and for each component category. The relative distribution of per-participant costs per component category for MAP-I and CBT-I are shown in the Fig. 1. Variable costs – those that vary with number of participants – were the highest participant cost category for MAP-I individually-delivered interventions, followed by time-dependent and then set-up costs. This ordering differed for group-delivered MAP-I interventions, for which variable costs were still the largest component and were then followed by set-up and time-dependent costs. For CBT-I, variable costs were also the largest component, followed by intervention setup and then time-dependent costs; this was consistent across both individually- and group delivered interventions.

Costs of all individual CBT-I components were higher than those for MAP-I except for instructor training course/exam costs, for which per-participant CBT-I costs were 4-fold lower; however, because CBT-I training time costs are almost 5-fold higher than those for MAP-I, total instructor training costs were still higher for CBT-I. For individually-delivered interventions, the 5 largest per-participant MAP-I cost components were: instructor in-class time ($594); caregiving time while participants traveled to/from and participated in the intervention ($402); participant time in sessions ($268); instructor training courses, exams ($224); and participant time in travel to/from interventions ($134). For individual- delivered interventions, the 5 largest per-participant CBT-I cost components were: instructor in-class time ($1802), caregiving time while participants traveled to/from and participated in the intervention ($426); instructor structured treatment planning time ($328); instructor training time ($320); and participant time in sessions ($293). MAP-I and CBT-I per-participant cost details for each component category are reported below.

### Intervention set-up costs

For individually-delivered programs, per-participant setup costs were $329 for MAP-I and $425 for CBT-I, resulting in a 23% cost savings with MAP-I. For group-delivered programs, these costs were $367 and $465, respectively, for a 21% MAP-I savings. For both MAP-I and CBT-I, instructor training costs were by far the largest component of set-up costs, followed by materials preparation and participant scheduling. For group-based programs, room setup and cleanup also contributed a large proportion of setup costs. Staff training was the lowest set-up cost.

### Time-dependent costs

For individually-delivered interventions, per-participant time-dependent costs were $665 for MAP-I and $2441 for CBT-I, for a $1776 (73%) MAP-I cost savings. For group-delivered programs, these costs were $121 and $404, respectively, for a $283 MAP-I (70%) savings. For both interventions delivered individually or in groups, instructor in-class time was the largest individual time-dependent cost component. Overhead and facilities were $0 for both interventions. The other lowest time-dependent cost components for MAP-I were those required for CBT-I only: staff time calculating sleep efficiency and instructor treatment planning time. For CBT-I, the other lowest time-dependent components were staff time in session tracking and calculating sleep efficiency.

### Variable costs

For individually-delivered interventions, per-participant variable costs were $890 and $1112 for MAP-I and CBT-I, respectively, for a 20% MAP-I savings. Because these costs vary with number of participants regardless of whether the intervention is delivered individually or in groups, they were the same for group-delivered interventions. The largest individual variable cost component was caregiving time while the participants travel to/from and participate in the intervention, followed by participant time in sessions and traveling to/from sessions. Variable equipment costs were the lowest variable cost component.

### Sensitivity analyses

Sensitivity analysis results are shown in Table 4. Per-participant costs for CBT-I were higher than those for MAP-I with all sensitivity analyses, with per-participant MAP-I savings for individually-delivered programs ranging from $2094 in the base case to $353 when all instructor time costs – the largest cost component – were excluded from the analysis. For group-delivered interventions, these savings range from $604 in the base case to $355 when excluding all instructor time. Participant travel time costs, which were the same between interventions, had no impact on MAP-I savings as compared with the base case. When indirect costs associated with participant time and other AD caregiver time were excluded to consider results from the US healthcare system perspective, MAP-I per-participant savings decreased to $1872 (65%) for individually-delivered and $382 (44%) for group-delivered interventions.

**Table 4:** Sensitivity analyses: MAP-I and CBT-I per-participant costs for individually- and group-based programs^a^

SENSITIVITY ANALYSIS	INDIVIDUALLY-DELIVERED PROGRAMS			GROUP-DELIVERED PROGRAMS		
	MAP-I	CBT-I	Savings with MAP-I	MAP-I	CBT-I	Savings with MAP-I
**Base case (societal perspective)**	**$1884**	**$3978**	**$2094**	**$1377**	**$1981**	**$604**
Healthcare system perspective^b^	$995	$2867	**$1872**	$488	$870	**$382**
Excluding costs of: ^c^						
Participant travel time	$1750	$3844	**$2094**	$1243	$1847	**$604**
Fixed equipment	$1883	$3972	**$2089**	$1376	$1975	**$599**
Caregiver time	$1482	$3552	**$2070**	$975	$1555	**$580**
Instructor training	$1594	$3607	**$2013**	$1087	$1610	**$523**
All participant time	$1397	$3293	**$1896**	$890	$1296	**$406**
Instructor non-structured time outside-of-sessions	$1843	$3732	**$1889**	$1371	$1946	**$575**
Booster sessions	$1214	$2476	**$1263**	$925	$1308	**$383**
Instructor structured time^d^	$1290	$1848	**$558**	$1292	$1677	**$385**
Instructor salary differences^e^	$1884	$2404	**$520**	$1377	$1780	**$403**
All instructor time	$1249	$1602	**$353**	$1287	$1642	**$355**

*CBT-I* Cognitive behavioral therapy for insomnia, *MAP-I* Mindful awareness practices for insomnia

^a^Individually-based programs assume that interventions are delivered to individuals in a 1:1 instructor-to-participant ratio; group-based programs assume group-based delivery in a 1:7 instructor-to-participant ratio

^b^Healthcare system perspective excludes participant and other AD caregiver time costs

^c^Listed in order of least-to-greatest impact on savings with MAP-I, considering individually-based programs

^d^Structured instructor time includes time both in sessions and in structured treatment plan development

^e^Assuming CBT-I instructors and MAP-I instructors all paid at MAP-I salary + benefits rate

When assuming individually-run programs, the following cost components represented the largest cost categories and had the greatest impact on MAP-I savings (shown in parentheses): all instructor time (structured and non-structured; $353); differences in MAP-I vs. CBT-I instructor salaries ($520); structured instructor time (in-session and in treatment planning; $558); booster sessions ($1263); and healthcare perspective (excludes all participant and other AD caregiver time costs; $1872).

When assuming group-run programs, the following cost components represented the largest cost categories and had the greatest impact on MAP-I savings (shown in parentheses): all instructor time (structured and non-structured; $355); healthcare perspective (excludes all participant and other AD caregiver time costs; $382); booster sessions ($383); structured instructor time (in-session and in treatment planning; $385); and differences in MAP-I vs. CBT-I instructor salaries ($403).

## Discussion

We conducted the first micro-costing analysis of an MBI for treating insomnia in AD caregivers. Our results demonstrate that substantial cost savings can be attained when providing MAP-I rather than CBT-I in this population, and these findings are robust across a range of scenarios. From the societal perspective, MAP-I and CBT-I were found to cost $1884 and $3978 per caregiver respectively when the interventions are delivered on an individual basis, and $1377 and $1981 respectively when delivered to small groups of caregivers. These results translate into cost savings associated with MAP-I vs. CBT-I of $2094 (53%) and $604 (30%) per AD caregiver treated individually or in a small group, respectively. From the perspective of the US healthcare system, these respective cost savings are $1872 (65%) and $382 (44%) per treated caregiver. Given that the non-inferiority trial of MAP-I vs. CBT-I is ongoing and insomnia outcomes for the two interventions are not yet known, we emphasize that the present findings are limited to a cost analysis and not a cost efficacy analysis.

These cost differences are primarily driven by the inherent complexity of the CBT-I approach, both in general and especially as compared with that of MAP-I. CBT-I requires more participant time, both in sessions and at home tracking and reporting program metrics. Instructor time is likewise greater, both with participants and reviewing their progress to develop individually-tailored treatment plans, which is necessary in the AD caregiver population given the heterogeneity in AD patient clinical status between caregivers, as well as over time within a caregiver and AD patient dyad. The cost implications of these time-resource differences are accentuated by the fact that CBT-I instructors are considered highly-trained and skilled therapists whose typical salary is over three times that of a MAP-I instructor. Although CBT-I could be delivered by providers with fewer certifications (e.g., without behavioral sleep medicine certification), this was not assumed for costing purposes in order to reflect the training attained by providers in the CARES clinical trial. Even if instructor training costs were minimal-to-none for CBT-I and MAP-I providers, sensitivity analyses indicate that MAP-I still provides savings over CBT-I. That said, overall costs associated with each program may lessen if providers with lesser training were to be used to roll out these programs in real-world settings. While treatment approaches such as digital CBT-I might also show lower costs than in-person or clinician-delivered CBT-I, prior non-inferiority trials have found that clinician-delivered CBT-I yields greater and more durable effects, with higher rates of adherence and retention, than digital CBT-I [36–38].

The inclusion in the CARES trial and this analysis of 5 monthly booster sessions after the 6-week treatment period also greatly impacted cost estimates. Although these sessions are likely to increase the level and duration of treatment effect, excluding them may better reflect real-world implementation. In such a scenario, costs of both interventions decreased by over one-third and MAP-I savings decreased to $1263 (51%) and $383 (29%) for individually- and group-delivered interventions, respectively.

Analyses such as these are critical for increasing implementation of MBIs in this era of ever-increasing healthcare costs and the need for low-cost and effective interventions. Strengths of this analysis include its robust methodologic approach, following the micro-costing guidelines of the US Panel on Cost Effectiveness [28]. Using detailed utilization and cost data, this analysis provides essential input for future economic analyses of these interventions. Collecting data alongside a clinical trial increase our findings’ validity and reliability, having allowed us to extract types, quantities, and costs of resources used as consumption occurred. The trial itself is significant in targeting insomnia in the AD caregiver population; evaluating a validated, curriculum-based, scalable self-care intervention tailored for insomnia treatment; and implementing strategies to encourage maintenance of practice over time. MAP-I savings are robust to all scenarios evaluated, and presenting results from both societal and healthcare system perspectives provides a broad policy understanding of the cost implications associated with insomnia treatment decision-making.

We recognize that limitations exist in conducting micro-costing analyses. Being a labor-intensive process, such a micro-costing approach may not be replicated in other studies of insomnia treatment among AD caregivers or other populations, preventing cost comparisons with other interventions. The cost estimates derived from this analysis may also not be generalizable to other settings with different costs for staff, therapists’, and project managers’ time, or when these interventions are conducted outside of a clinical trial setting. However, given that resources needed for these interventions and the MAP-I and CBT-I instructor salary differences are expected to be relatively consistent across settings, it is likely that a similar measure of savings will be found across settings. If a macro-costing, or top-down, approach were to be used instead of the micro-costing, bottom-up approach taken here, differences in payer assumptions would be highlighted and cost differentials would likely vary more greatly between settings. Patient-borne costs were not considered in this analysis, and would likely also differ between settings based not only on the number of sessions but also on payer assumptions regarding each intervention’s costs. Intervention-related changes in caregiver healthcare utilization were not collected in the CARES trial and their associated costs were not included in this analysis. Although this could have resulted in our over-estimating net MAP-I and CBT-I costs, it is expected that such utilization changes would be similar between interventions and thus not impact the general conclusions of MAP-I savings.

Despite these limitations, it is well understood that measuring resources used in medical and public health programs is important for understanding the overall and economic implications of such interventions. Saving approximately $2000 per person may seem trivial relative to the annual $3.6 trillion spent on healthcare in the US [39]. However, that $3.6 trillion equates to approximately $11,000 per person, of which a $2000 savings would not be inconsequential. If we further consider that 60% of the 5 million US AD caregivers have insomnia, saving $2094 per treated caregiver would provide a total societal cost savings of $6.3 billion per year. The opportunity costs of leaving these potential savings on the table are far-reaching and cannot be ignored.

It is hypothesized that results of the ongoing non-inferiority CARES trial will demonstrate that MAP-I is of non-inferior efficacy to CBT-I, given the magnitude of the MAP-I treatment effect on sleep quality in older adults as previously reported [24]. With the potential for cost savings offered by MAP-I, the results of this analysis and the CARES trial will be critical for policymakers to consider in reevaluating the guideline-recommended treatment for insomnia. Insomnia is among the most prevalent behavioral symptoms in AD caregivers, adds to caregiving burden and is associated with reduced health across a range of conditions [2]. Incorporating MAP-I as a viable alternative in these guidelines would substantially increase treatment options and access, representing a significant and necessary paradigm shift for this vulnerable population. Barriers related to MAP-I intervention uptake, adherence, and dissemination are small, with its simple at-home approach, its ongoing implementation in various settings, and the availability of an on-line version. Given the increasing growth of the aging population and predicted financial cost of chronic disease burden [2], improving insomnia treatment options and access has real-world implications for reducing morbidity and mortality in this vulnerable AD caregiver population.

## Conclusions

This novel analysis demonstrates that substantial cost savings can be attained when providing MAP-I instead of CBT-I as insomnia treatment for the AD caregiver population. Micro-costing methods such as those implemented here are critical for guiding decision-making among policy-makers, providers, and patients alike. These findings will help increase dissemination and adoption of these interventions and thereby improve insomnia, health, and aging outcomes among this important and large AD caregiver population.

## Supplementary Information

**Additional file 1.**

**Additional file 2.**

## Data Availability

All data supporting the conclusions of this article are fully available without restriction and are included within the manuscript and its supporting information files.

## Abbreviations

AD: Alzheimer’s disease
CARES: Caregiver Sleep Research
CBT-I: Cognitive behavioral therapy for insomnia
FTE: Full-time equivalent
MAP-I: Mindful awareness practices for insomnia
MBI: Mindfulness-based intervention
N/A: Not applicable
UCLA: University of California Los Angeles
US: United States
