# Supplementary material for: Costs associated with treatment of insomnia in Alzheimer’s disease caregivers: a comparison of mindfulness meditation and cognitive behavioral therapy for insomnia
Source: BMC Health Serv Res. 2022 Feb 19;22:231. doi: 10.1186/s12913-022-07619-w (PMC8858547; doi:10.1186/s12913-022-07619-w)
Supplement: Supplementary file 1 — Additional file 1. [file 12913_2022_7619_MOESM1_ESM.docx]

**Supplemental Table 1a. MAP-I instructor training components and costs**

| **Training Components** | **Cost per hour*** | **Costs per component** | **Total Cost** |
| --- | --- | --- | --- |
| 1 MAP class (6 weeks x 2 hours/week) | Flat rate | $200 | $200 |
| 1 year practice 20 mins/day | $27.01 | $9 | $3,286 |
| 2 week-long retreats | Flat rate | $2,000 | $4,000 |
| Training in Mindfulness Facilitation Course (16, 8-hour days facilitator training over 1 year) | Flat rate | $7,000 | $7,000 |
| **Total Cost** |  |  | **$14,486** |

mins, minutes

* Average hourly wage for MAP-I instructors based on UCLA salaries.

**Supplemental Table 1b. CBT-I Instructor Training Components and Costs^*^**

| **Training Component** | **Cost per hour**** | **Minimum** | | **Moderate** | | | | **Maximum** | | | |  | |  |
| --- | --- | --- | --- | --- | --- | --- | --- | --- | --- | --- | --- | --- | --- | --- |
|  |  | **# hours** | **Cost** | **# hours** | | **Cost** | | **# hours** | | **Cost** | | **Average** | |  |
| Basic Course | | | | | | | | | | | | | |  |
| Course Purchase |  | Flat rate | $600 | | Flat rate | | $600 | | Flat rate | | $600 | | $600 | |
| Manual |  | Flat rate | $35 | | Flat rate | | $35 | | Flat rate | | $35 | | $35 | |
| DVDs, Optional Materials |  | N/A | $0 | | N/A | | $0 | | Flat rate | | $500 | | $167 | |
| Study Time | $81.92 | 10 | $819 | | 20 | | $1,638 | | 35 | | $2,867 | | $1,775 | |
| Additional Training | | | | | | | | | | | | | |  |
| Supervised behavioral sleep medicine work† | $81.92 | 0 | $0 | | 150 | | $12,288 | | 250 | | $20,481 | | $10,923 | |
| Supervisor/ consultant time† | $150.00 | 0 | $0 | | 7 | | $1,050 | | 7 | | $1,050 | | $700 | |
| Advanced Course |  | N/A | $0 | | N/A | | $0 | | Flat rate | | $600 | | $200 | |
| Mini-Fellowship |  | N/A | $0 | | N/A | | $0 | | Flat rate | | $1,724 | | $575 | |
| Exam | | | | | | | | | | | | | |  |
| Prep | $81.92 | 40 | $3,277 | | 40 | | $3,277 | | 40 | | $3,277 | | $3,277 | |
| Exam Purchase |  | Flat rate | $250 | | Flat rate | | $250 | | Flat rate | | $250 | | $250 | |
| **Total Cost** |  | **$4,981** | | | **$19,139** | | | | **$31,384** | | | | **$18,501** | |

* Because CBT-I instructor training comprises some components that are optional for certification, costs were estimated considering a range of minimum, moderate, and maximum level training approaches and, assuming that these approaches would be implemented with equal likelihood across instructors, the average costs of all approaches was estimated and used for this analysis.

** Average hourly wages for CBT-I therapists based on UCLA salaries; and for supervisors/consultants, based on expert guidance provided January 2020 via email from Donn Posner, a CBT-I expert at Stanford School of Medicine.

† To sit for the exam, the Society of Behavioral Sleep Medicine recommends 250 hours of behavioral sleep medicine work under consultation with an expert, with at least 1 hour/week of supervisor consultation; assuming 40-hour weeks equates with a minimum of 6.25 direct supervision cases, which are rounded up to 7 to avoid person-divisibility.
