# Supplementary material for: Costs associated with treatment of insomnia in Alzheimer’s disease caregivers: a comparison of mindfulness meditation and cognitive behavioral therapy for insomnia
Source: BMC Health Serv Res. 2022 Feb 19;22:231. doi: 10.1186/s12913-022-07619-w (PMC8858547; doi:10.1186/s12913-022-07619-w)
Supplement: Supplementary file 2 — Additional file 2. [file 12913_2022_7619_MOESM2_ESM.docx]

#### Supplemental Table 2a. MAP-I and CBT-I resource utilization: intervention set-up hours per program, group, and participant^*^

| **Component** | | **Human Resource** | **Notes** | | **MAP-I** | | | | | | **CBT-I** | | | | | |
| --- | --- | --- | --- | --- | --- | --- | --- | --- | --- | --- | --- | --- | --- | --- | --- | --- |
|  |  |  |  |  | **Program** | | **Group** | | **Participant** | | **Program** | | **Group** | | **Participant** | |
| **General Session Set-Up** | | | |  | |  | |  | |  | |  | |  | |  |
|  | Session planning | RA | 1 hr per group | | 1 | | 1 | | 0 | | 1 | | 1 | | 0 | |
|  | Materials, handouts prep | RA | 30 mins. per session x 6 sessions | | 3 | | 3 | | 0.42 | | 3 | | 3 | | 0.42 | |
|  | Participant scheduling | RA | 1 hr per group | | 1 | | 1 | | 0.14 | | 1 | | 1 | | 0.14 | |
|  | Room setup, cleanup^**^ | RA | 30 mins. per session x 6 sessions | | 3 | | 3 | | 0 | | 3 | | 3 | | 0 | |
| **Makeup Sessions Set-Up**^†^ | | | |  | |  | |  | |  | |  | |  | |  |
|  | Session planning | RA | 0.5 hrs per session | | 0.5 | | 0.29 | | 0.04 | | 0.5 | | 1.21 | | 0.17 | |
|  | Participant scheduling | RA | 0.5 hrs per session | | 0.5 | | 0.29 | | 0.04 | | 0.5 | | 1.21 | | 0.17 | |
|  | Room setup, cleanup^**^ | RA | 15 mins. per session | | 0.25 | | 0.14 | | 0 | | 0.25 | | 0.61 | | 0 | |
| **Booster Sessions Set-Up** | | | |  | |  | |  | |  | |  | |  | |  |
|  | Session planning | RA | 0.5 hrs per session x 5 sessions | | 2.5 | | 2.5 | | 0 | | 2.5 | | 2.5 | | 0 | |
|  | Materials, handouts preparation | RA | 0.5 hrs per session x 5 sessions | | 2.5 | | 2.5 | | 0.35 | | 2.5 | | 2.5 | | 0.35 | |
|  | Participant scheduling | RA | 1 hr per group | | 1 | | 1 | | 0.14 | | 1 | | 1 | | 0.14 | |
|  | Room setup, cleanup^**^ | PM | 15 mins. per session x 5 sessions | | 1.25 | | 1.25 | | 0 | | 1.25 | | 1.25 | | 0 | |

CBT-I, cognitive behavioral therapy for insomnia; hrs, hours; MAP-I, mindful awareness practices for insomnia; mins, minutes; PM, project manager; RA, research assistant.

* Values reported in number of hours.

** Per-participant time is zero for room set-up/cleanup, assuming that individually-delivered interventions do not require rooms outside of instructor/therapists' office.

#### ^†^ The trial reported 4.2 times more makeup sessions in the CBT-I arm than MAP-I.

#### Supplemental Table 2b. MAP-I and CBT-I resource utilization: time-dependent hours per program, group, and participant*

| **Component** | | **Human Resource** | **MAP-I** | | | | | | **CBT-I** | | | | | | | |  |  |
| --- | --- | --- | --- | --- | --- | --- | --- | --- | --- | --- | --- | --- | --- | --- | --- | --- | --- | --- |
|  |  |  | **Notes** | **Program** | | **Group** | **Participant** | | **Notes** | | **Program** | | **Group** | | **Participant** | | | |
| **General Session Management** | | | | | | | | | | | | | | |  | | |  |
|  | Materials distribution | RA | 15 mins. per session x 6 sessions | | 1.5 | 1.5 | 0.21 | 15 mins. per session x 6 sessions | | 1.5 | | 1.5 | | 0.21 | |  |  |  |
|  | Session tracking | RA | 15 mins. per session x 6 sessions | | 1.5 | 1.5 | 0.21 | 15 mins. per session x 6 sessions | | 1.5 | | 1.5 | | 0.21 | |  |  |  |
|  | Calculating sleep efficiency, planning tx | RA | N/A | | 0 | 0 | 0 | 45 mins. per session x 6 sessions | | 4.5 | | 4.5 | | 0.63 | |  |  |  |
| **Makeup Session Management** | | | | | | | | | | | | | | | | | |  |
|  | Materials distribution | RA | 15 mins. per session x 6 sessions | | 1.5 | 0.86 | 0.12 | 15 mins. per session x 6 sessions | | 1.50 | | 3.64 | | 0.51 | |  |  |  |
|  | Session tracking | RA | 10 mins. per session | | 0.17 | 0.10 | 0.01 | 10 mins. per session | | 0.17 | | 0.40 | | 0.06 | |  |  |  |
|  | Calculating sleep efficiency, planning tx | RA | N/A | | 0 | 0 | 0 | 30 mins. per session | | 0.50 | | 1.21 | | 0.17 | |  |  |  |
| **Booster Session Management** | | | | | | | | | | | | | | | | | |  |
|  | Materials distribution | RA | 15 mins. per session x 5 sessions | | 1.25 | 1.25 | 0.18 | 15 mins. per session x 5 sessions | | 1.25 | | 1.25 | | 0.175 | |  |  |  |
|  | Session tracking | RA | 15 mins. per session x 5 sessions | | 1.25 | 1.25 | 0.18 | 15 mins. per session x 5 sessions | | 1.25 | | 1.25 | | 0.175 | |  |  |  |
|  | Calculating sleep efficiency, planning tx | RA | N/A | | 0 | 0 | 0 | 30 mins. per session x 5 sessions | | 3 | | 3 | | 0.42 | |  |  |  |
| **Instructor Time** | | | | | | | | | | | | | | | | | |  |
|  | Regular session in-class time | Instructor/ therapist | 2 hrs. x 6 sessions | | 12 | 12 | 12 | 2 hrs. x 6 sessions | | 12 | | 12 | | 12 | |  |  |  |
|  | Structured treatment plan development (regular) | Instructor/ therapist | N/A | | 0 | 0 | 0 | Two 1-hr sessions | | 2 | | 2 | | 2 | |  |  |  |
|  | Non- structured time with participants | Instructor/ therapist | 15 min. x 6 sessions | | 1.5 | 1.5 | 1.5 | 30 min. x 6 sessions | | 3 | | 3 | | 3 | |  |  |  |
|  | Booster session in-class time | Instructor/ therapist | 2 hrs. x 5 booster sessions | | 10 | 10 | 10 | 2 hrs. x 5 booster sessions | | 10 | | 10 | | 10 | |  |  |  |
|  | Structured treatment plan development (boosters) | Instructor/ therapist | N/A | | 0 | 0 | 0 | Two 1-hr sessions | | 2 | | 2 | | 2 | |  |  |  |

CBT-I, cognitive behavioral therapy for insomnia; hrs, hours; MAP-I, mindful awareness practices for insomnia; mins, minutes; N/A, not applicable; PM, project manager; RA, research assistant; tx, treatment

* Values reported in number of hours.

#### Supplemental Table 2c. MAP-I and CBT-I resource utilization: variable resource hours per program, group, and participant*

| **Component** | | **Human Resource** | **MAP-I** | | | | **CBT-I** | | | |
| --- | --- | --- | --- | --- | --- | --- | --- | --- | --- | --- |
|  |  |  | **Notes** | **Program** | **Group** | **Participant** | **Notes** | **Program** | **Group** | **Participant** |
| **Caregiver Time** | | |  |  |  |  |  |  |  |  |
|  | Caregiving while participant at intervention | hired caregiver | 12 hrs regular sessions, 10 hrs boosters, 12 hrs travel | 33 | 236 | 33 | 14 hrs regular sessions, 10 hrs boosters, 12 hrs travel | 35 | 250 | 35 |
| **Participant Time** | | |  |  |  |  |  |  |  |  |
|  | In regular sessions | participant | 2 hr. session x 6 sessions | 12 | 86 | 12 | 2 hr. session x 6 sessions + two 1-hr 1:1 sessions | 14 | 100 | 14 |
|  | In booster sessions | participant | 2 hr. session x 5 booster sessions | 10 | 71 | 10 | 2 hr. session x 5 booster sessions | 10 | 71 | 10 |
|  | Travel to/from 6 regular sessions | participant | 1 hr avg travel x 6 sessions | 6 | 43 | 6 | 1 hr avg travel x 6 sessions | 6 | 43 | 6 |
|  | Travel to/from 5 booster sessions | participant | 1 hr avg travel x 5 sessions | 5 | 36 | 5 | 1 hr avg travel x 5 sessions | 5 | 36 | 5 |
|  | Completing daily sleep diaries, regular sessions | participant | N/A | 0 | 0 | 0 | 10 mins. x 7 days x 6 weeks | 7 | 50 | 7 |
|  | Completing daily sleep diaries, booster sessions | participant | N/A | 0 | 0 | 0 | 10 mins. x 7 days x 6 weeks | 7 | 50 | 7 |
|  | Calling in sleep diaries, regular sessions | participant | N/A | 0 | 0 | 0 | 5 mins. x 7 days x 6 weeks | 3.5 | 25 | 3.5 |
|  | Completing "worry log," regular sessions | participant | N/A | 0 | 0 | 0 | 5 mins. for 1 week | 0.08 | 0.60 | 0.08 |
|  | Calling in sleep diaries, booster sessions | participant | N/A | 0 | 0 | 0 | 5 mins. x 7 days x 6 weeks | 3.5 | 25 | 3.5 |
|  | Completing "worry log," booster sessions | participant | N/A | 0 | 0 | 0 | 5 mins. for 1 week | 0.08 | 0.60 | 0.08 |
|  | Home meditation practice | participant | 30 mins. x 6 weeks | 3 | 21.43 | 3 | N/A | 0 | 0 | 0 |
|  | Completing meditation practice diaries, regular sessions | participant | 5 mins. x 7 days x 6 weeks | 3.5 | 25 | 3.5 | N/A | 0 | 0 | 0 |
|  | Completing meditation practice diaries, booster sessions | participant | 5 mins. X 5 booster sessions | 0.42 | 2.98 | 0.42 | N/A | 0 | 0 | 0 |

Avg, average; CBT-I, cognitive behavioral therapy for insomnia; hrs, hours; MAP-I, mindful awareness practices for insomnia; mins, minutes; N/A, not applicable; tx, treatment

* Values reported in number of hours.
